# Bone Marrow Vascular Niche: Home for Hematopoietic Stem Cells

**DOI:** 10.1155/2014/128436

**Published:** 2014-04-14

**Authors:** Ningning He, Lu Zhang, Jian Cui, Zongjin Li

**Affiliations:** ^1^Department of Pathophysiology, Nankai University School of Medicine, 94 Weijin Road, Tianjin 300071, China; ^2^The Key Laboratory of Bioactive Materials, The College of Life Science, Nankai University, Ministry of Education, Tianjin 300071, China; ^3^Department of Intensive Care Unit (ICU), People's Hospital of Rizhao, Shandong 276826, China

## Abstract

Though discovered later than osteoblastic niche, vascular niche has been regarded as an alternative indispensable niche operating regulation on hematopoietic stem cells (HSCs). As significant progresses gained on this type niche, it is gradually clear that the main work of vascular niche is undertaking to support hematopoiesis. However, compared to what have been defined in the mechanisms through which the osteoblastic niche regulates hematopoiesis, we know less in vascular niche. In this review, based on research data hitherto we will focus on component foundation and various functions of vascular niche that guarantee the normal hematopoiesis process within bone marrow microenvironments. And the possible pathways raised by various research results through which this environment undergoes its function will be discussed as well.

## 1. Introduction

Human hematopoietic stem cells (HSCs) firstly appear in the earliest embryo, then move to fetal liver and spleen, and ultimately migrate to bone marrow (BM). Self*-*renewal and differentiation of HSCs are tightly regulated by both cell intrinsic and extrinsic factors and a lifelong homeostasis of functional HSCs is achieved [1–3]. It is hypothesized that there must be specific microenvironments existing within the BM area that contain HSCs and other supporting cells, organizing interaction between cells and cells and cells and factors in order to sustain specific aspects of hematopoiesis, such as HSC survival, self-renewal, and differentiation. These processes have been linked to a number of different stromal cell types and signaling pathways. BM has a homogenous architecture devoid of structural and functional partitions and the periphery of adult BM cavity harbors compartments with distinct properties [4]. Recent study revealed that HSCs preferentially localize in endosteal zones, where they most closely interact with sinusoidal and nonsinusoidal bone marrow microvessels, which form a distinctive circulatory system [5]. These special hematopoietic microenvironments are also termed as “Niche,” which was first proposed by Schofield in 1978 to describe areas where HSCs reside in [6].

According to current hypothesis, the specialized region within BM that HSCs dwell into has been classified into two types: osteoblastic niche and BM vascular niche [7, 8]. HSCs are mainly maintained by the endosteal osteoblast niche, which provides a quiescent HSC microenvironment. And the vascular niche, can regulate the proliferation, differentiation, and mobilization of HSCs [9]. Nonetheless, developments in this field will be useful for the advancement of HSC expansion and transplantation in the future.

## 2. HSCs Interaction with Specialized Microenvironments

Back to early 1970s, undifferentiated hematopoietic cells were indicated to localize close to the endosteal bone surface [10, 11], and an increase of osteoblasts number was accompanied with increased BM HSCs* in vivo* [12]. And subsequent investigators proposed the concept of “niche.” The main task burdened on osteoblastic niche is to maintain the long-term HSCs (LT-HSCs), which are capable of supporting hematopoiesis for months or even a lifetime as quiescent or low-cycling cells [7, 13–15]. It is revealed that an increase in the number of osteoblastic lining cells in the trabecular bone area correlates with increase in the number of LT-HSCs [13]. However, since maintaining HSCs is just the headstream of hematopoiesis, the rest sections of hematopoietic processes within BM, such as mobilization, migration, and differentiation of HSCs, are also significant for hematopoietic research. This is why the newly discovered vascular niche draws researchers' attention on its crucial role in blood system.

### 2.1. The Model of HSCs Niche

**Figure 1:**
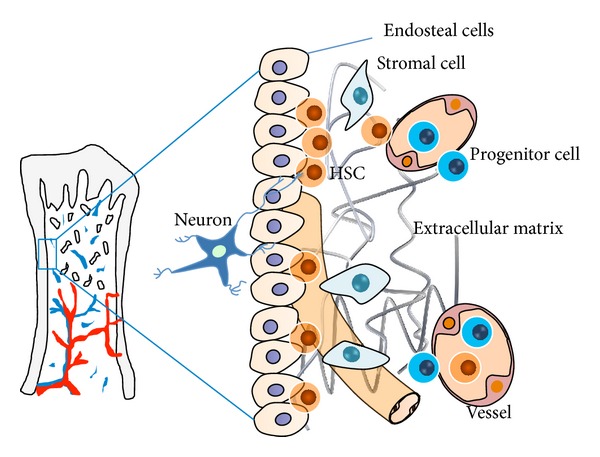
The model of HSCs niche. The hematopoietic niches are where hematopoietic stem cells (HSCs) mainly reside in during adulthood. HSCs niche is composed of complex components including HSCs and other functional elements such as vessels, stromal cells, ECM proteins, neural inputs, and endothelial cells (ECs). Only through interaction with these components HSCs can keep self-renewal and differentiation. Under the help of such “blocks,” the progenitor cells derived from HSCs locating at the inner surface of BM migrate to blood vessels at the center of the BM cavity when they differentiate into mature blood cells.

Studies on* Drosophila, C. elegans,* and many mammals have revealed critical features of stem cell niches by means of signals emanating from certain cells and adhesion between stem cells and supporting stromal cells or extracellular matrix (ECM), which have the ability to direct the self-renewal, survival, maintenance, and differentiation of stem cells [16, 17]. In order to operate such functions, complex components of niches are foundational blocks that set up such “micropalace.” By integrating data from numerous systems, we can generate a hypothetical “parts list” for HSC niches, including the HSC itself; stromal cells; ECM proteins; blood vessels; neural inputs; and endothelial cells (ECs) (Figure 1). The interactions of HSC-niche influence the self-renewal [7, 13], quiescence [18–20], or mobilization of HSCs [21], depending on multiple intrinsic and extrinsic factors.

### 2.2. Interaction of Different Components in HSCs Niche

By interactions with these “blocks” of HSC niches, the progenitor cells derived from HSCs locating at the inner surface of BM migrate to blood vessels at the center of the BM cavity and then differentiate into mature cells. HSCs were hypothesized to be adhered to the surface of osteoblasts based on the observation of N-cadherin^+^ cells at the endosteum that express some HSC markers [13, 14, 22]. By producing regulators such as angiopoietin [14] and osteopontin [18], they are able to regulate HSC pool size* in vivo*. The osteoclasts are also important components, which are essential for bone resorption. Activation of osteoclasts also reduces the stem cell niche components, SDF-1 and SCF, which leads to subsequent mobilization of HSCs [23]. The form of bone is regulated by osteoblasts and osteoclasts in dynamic equilibrium under steady-state conditions [24, 25], influencing the destiny of HSCs. What is more, since the endosteal surface is heavily vascularized, vascular and perivascular cells, such as reticular cells, adipocytes, and mesenchymal progenitors, might contribute to the formation of HSC niches at or near the endosteum [26, 27]. Along with osteoblastic niche, this type of microenvironment was defined as “vascular niche” [8]. And further experiment confirmed that exposing HSCs to endothelial cells (ECs) isolated from different tissues results in varying HSC growth and repopulating ability [28].

Perivascular CXCL12-abundant reticular cells (CAR cells) were shown to be necessary for maintenance of BM HSC content* in vivo *[26]. On the contrary, BM adipocytes have negative effect on HSC pool size and function [29]. Most recently, it was demonstrated that nestin-expression MSCs (NES^+^ MSCs) are tightly related to the maintenance of HSCs and can breed osteoblasts in the BM HSC niche [30]. Thus, according to what has been mentioned above, it is likely that the cowork of bone cells, hematopoietic cells, and ECs in the osteoblastic niche regulates hematopoiesis.

## 3. Various Roles of Vascular Niche in Hematopoiesis

**Figure 2:**
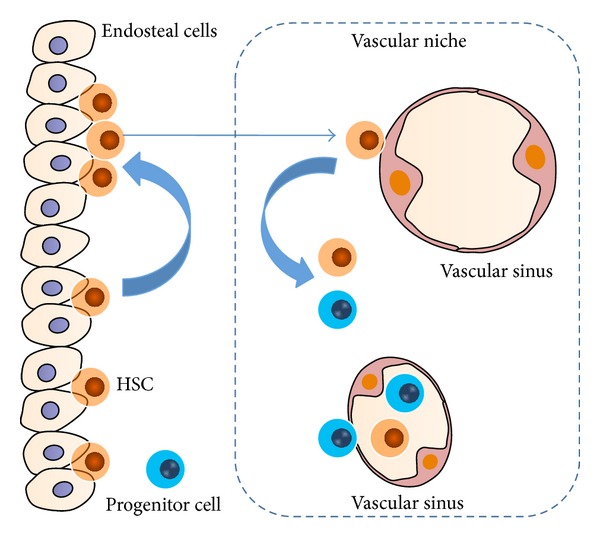
Vascular niche plays a key role in hematopoiesis. Vascular niche is termed as an alternative niche compared to osteoblastic niche. Through the interaction between the HSCs and ECs, HSCs operate the process of self-renewal and differentiation. The hematopoietic progenitor cells derived from HSCs quiescent in osteoblastic niche penetrate the ECs to reside in vascular niche and then differentiate into different kinds of blood cells, entering the circulation system.

The research about vascular niche made a breakthrough much later than what has been done for osteoblastic niche. What is clear is that, through the interaction between the HSCs and ECs, HSCs operate the processes of self-renewal and differentiation [31–33]. The hematopoietic progenitor cells derived from HSCs quiescent in osteoblastic niche penetrate the ECs to reside in vascular niche, then differentiate into different kinds of blood cells, and ultimately enter into the circulation system [32, 34, 35] (Figure 2).

### 3.1. Cellular Foundation of Vascular Niche

The cellular foundation in vascular niche is heterogeneous in its nature and origin: sinusoidal vessels are supplied by arterioles and capillaries which are derived from the furcation of arterial vessels spanning the marrow cavity [32]. The sinusoids are interconnected by intersinusoidal capillaries and collectively drain into the central sinus. In addition to providing a niche for the self-renewal, expansion, and maintenance of HSCs, sinusoidal endothelial cells (SECs) play a role both in providing a differentiation platform for hematopoietic cells and a conduit for mobilization and homing hematopoietic cells into and out of the BM [32, 36]. The idea that sinusoids may represent a proliferative niche is consistent with recent studies indicating that E-selectin, an adhesion molecule constitutively expressed in certain bone marrow sinusoidal microdomains, promotes HSCs proliferation and blockade of E-selectin protects HSCs following chemotherapy or *γ*-irradiation [37, 38]. In summary, discontinuous SECs constitute the functional hematopoietic vascular niche and are essential in the hematopoietic processes. Coacting with HSCs and their surrounding, vascular niche supports the series of hematopoietic processes when HSCs have been activated.

The histochemical analyses of the signaling lymphocyte activation molecule (SLAM) presented the results that about 60% of SLAM marked HSCs were visualized near the SECs indicating the existence of an alternative niche in the vascular zone [8, 39]. It was showed that, after BM transplantation, HSCs preferred to engraft in BM vascular domains in mice that received no other treatments [40].

### 3.2. Function of Vascular Niche

Up to now, it has been known that vascular niche takes effect in hematopoiesis and maintenance of HSCs. During hematopoiesis, HSCs are mobilized from dormant style [32, 41], migrate to bloodstream by penetrating the sinusoidal wall [35], and at last differentiate into several kinds of blood cells [42]. Once the BM was under stress, extramedullary hematopoiesis within vascular niche occurs in the spleen or liver to supplement the function in abnormal BM [43]. Along the process, there are various factors involved, which will be discussed as follows.

Moreover, endothelial cells also play an important role during hematopoietic regeneration showed by other studies. For example, the transplantation of endothelial progenitor cells speeded up the recovery of BM sinusoidal vessels after irradiation, correlated by higher recoveries of BM cellularity and HSCs [44].

In the spleens of cyclophosphamide/G-CSF-mobilized mice, about 62% HSCs with distinct surface marker CD150^+^CD48^−^CD41^−^ were observed in contact with sinusoidal ECs while the rest cells (38%) were always near sinusoids [4]. In another study, in close contact with liver sinusoid ECs, the extramedullary hepatic hematopoiesis occurs showing that liver provides similar microenvironment for maintenance and growth of hematopoietic cells compared with BM [8, 45, 46]. All these data propose that vascular niche may act as reinforcements to support the maintenance and differentiation of HSCs when the BM works abnormally.

### 3.3. Interaction with Osteoblastic Niche

As the sinusoid ECs act as an alternative HSCs niche along with osteoblastic niche, a proposal was raised that the vascular niche undergoes differentiation of HSCs while the osteoblastic niche offers a quiescent microenvironment [32]. This can be explained by the fact that the vascular niche compared with osteoblastic niche provides a more nutrient-rich microenvironment marked by higher concentrations of oxygen and growth factors, leading HSCs to be proliferative and mature blood cells, ultimately releasing into the peripheral circulation [32, 41]. Following this process another plausible function for this type of niche is to support HSCs in transendothelial migration. Blood cells can go through the sinusoidal wall easily because it consists of a single layer of ECs [35]. Therefore, not only can the vascular niche promote HSCs to proliferate and differentiate under hematopoietic stress [42], but also it enables immediate release of HSCs into the bloodstream.

Generally, the osteoblastic niche can maintain HSCs quiescence [14, 47]; meanwhile, the vascular niche regulates the mobilization of HSCs. However, it showed that ECs interacting with HSCs could play a necessary part of maintenance or proliferation of HSCs* in vitro* or* in vivo*. And since many HSCs in contact with BMECs are proliferating at any given time, it is probable that HSCs within the vascular bone-marrow are self-renewing, rather than long-term dormant [8]. Brandt and coworkers reported that porcine microvascular ECs (PMVECs) could mediate human HSCs expansion* ex vivo* and the HSCs isolated from PMVEC coculture were capable of repopulation in BM of SCID mice [48].* In vivo*, host-origin hematopoiesis could be restored in lethally irradiated mice after transplanting ECs isolated from the brain or lung, indicating ECs' essential role in the self-renewal and repair of HSCs [33]. Allogeneic endothelial progenitor cell infusions brought about hematopoietic reconstitution and an increase in LT-HSCs in mice with total body irradiation- (TBI-) induced myelosuppression, suggesting that ECs positively regulated HSC regeneration* in vivo* [44].

Furthermore, it was reported that hematopoietic cells may origin from ECs. During early development the hematopoiesis takes place along with vascularization and endothelium is responsible for HSCs emergence within the aorta-gonad-mesonephros (AGM) region* in vivo* [49]. Besides, many studies demonstrated that some cells in hematopoietic clusters, which are on the floor of the dorsal aorta in species ranging from amphibians through to humans, tightly are in contact with ECs in a disrupted region of lumen [49–51]. Subsequently, via lineage tracing experiments in both mice and zebrafish it is clear that hemogenic endothelium within the ventral aspect of the dorsal aorta is the original source of HSCs [52–56]. Thus, these elegant data point out that most HSCs activity has a close relationship with ECs, and endothelium within AGM is the original source of HSCs. Then the HSCs from AGM differentiated in fetal liver and finally settle down in adult BM [52, 57]. Therefore, endothelium performs like a factory, carrying on the ability to produce HSCs in the early development of organism. It is without impossibility that endothelium isolated from AGM can cure hematological disease without the presence of implanted HSCs.

## 4. Molecular Foundation for Vascular Niche Regulating the Hematopoiesis

The hematopoiesis regulated by the vascular niche refers to the functions that have been discussed above, including motility, transendothelial migration, hematopoietic differentiation, and maintenance of HSCs inside or outside the BM. And the relevant component surrounding HSCs is also included. So, a question is under pressing: through what mechanism or molecular foundation does this type of niche connect the HSCs and their surrounding to keep its regular work about hematopoiesis in order? The molecules regulating the hematopoiesis have been summarized in Table 1.

### 4.1. Stromal Cell-Derived Factor-1 (SDF-1)

It has been determined that stromal cell-derived factor-1 (SDF-1) expressed by BM ECs can stimulate HSCs and BM ECs, leading to an enhancement in transendothelial and stromal migration via activation of adhesion molecules [58, 59]. The process that the SDF-1 induces the transendothelial migration might depend on the immobilized chemokine fraction bound to components of the extracellular matrix (ECM) and BM stromal cells, named “haptotactic gradient” [60, 61]. Generally, quiescent HSCs trapped in osteoblastic niche must be firstly mobilized if hematopoiesis is needed. There was evidence showing that SDF-1 expressed by osteoblasts helps the HSCs homing to the osteoblast niche [62]. However, the expression of SDF-1 on ECs anticipates the key procedure in mobilization of HSCs.

Indeed, several reports indicate that SDF-1 plays a crucial role in maintaining HSC function, including retention in the bone marrow, quiescence, and repopulating activity. However, it is also well known that within the bone marrow SDF-1 is mainly expressed by three perivascular stromal cell populations: SDF-1-abundant reticular cells, nestin-stromal cells, and leptin receptor+ stromal cells. Although bone marrow endothelial cells also express SDF-1, the deletion of SDF-1 in specific niche cells indicates that endothelial cells are only minor contributors, and mesenchymal progenitors are the major source of SDF-1 that supports HSCs. In keeping with the notion that multiple niche constituents exert distinct functions, conditional deletion of Cxcl12 by Lepr-credoes does not affect HSC numbers in the bone marrow but induces their mobilization [63].

*In vitro*, in the presence of SDF-1 expressed by BMECs, the fragmentation of megakaryocytes is increased when CXCR-4- (SDF-1 receptor-) positive megakaryocytes migrate through a layer of BMECs, suggesting that the contact between megakaryocytes and BMECs is important for thrombopoiesis* in vitro* [36, 64]. Thus, SDF-1 is able to increase the affinity and migratory capacity of megakaryocytes across BMECs. Besides, endothelial FGF- (fibroblasts growth factor-) 4 showed to support the adhesion of megakaryocytes to BMECs. SDF-1 and FGF-4 have been reported to induce the expression of adhesion molecules, including very late antigen- (VLA-) 4 expressed by megakaryocytes and VCAM-1 expressed by BMECs [34, 65]. Similarly, the CXCR-4 antibody abrogates thrombocytosis bounce back after 5-FU treatment in c-Mpl^−/−^ mice and leads to decrease of megakaryocytes as well as a depleted vascular niche [36], which demonstrates that SDF-1 plays a key role in HSCs differentiation. Moreover, SDF-1 can promote vascular-dependent thrombopoiesis in thrombopoietin (TPO) deficient mice [36]. Since ECs are also marked by SDF-1, possibility that the mechanism of ECs directing the self-renewal of HSCs has relevant to SDF-1 remains, yet the molecular approach is under study.

Based on the mechanism of action of granulocyte colony stimulating factor (G-CSF), it was hypothesized that a gradient shift of different types of SDF-1 can cause HSC mobilization. The mobilization can be enhanced by SDF-1 on ECs promoting the hematopoietic progenitors to penetrate endothelium, resulting in differentiation. So, the mobilization induced by G-CSF is accompanied by a gradual decrease in osteoblastic SDF-1 and increase in CXCR- (endothelial SDF-1 receptor-) 4 expression on HSCs [66]. Through the similar mechanism, Tie2 (the endothelial receptor for angiopoietin-1 and angiopoietin-2) expression is reduced in the BM vascular niche during steady-state hematopoiesis. HSCs are also marked by Tie2, which mediates HSCs to adhesion to osteoblasts, regulating the quiescence in osteoblastic niche [14]. And inhibition of Tie2 signaling on ECs resulted in impaired reconstruction of the vascular niche as well as in delayed hematopoietic recovery [67]. Thus, Tie2 does something with maintenance of HSCs and the downregulation of Tie2 by G-CSF directs the motility of HSCs [14].

### 4.2. Notch Signaling

Moreover, a recent research demonstrated that ECs making the long-term HSCs undergo self-renewal potential have great relevance with Notch signaling [68]. Endothelial cells express the Notch ligands Jagged that support long-term HSC proliferation and prevent their exhaustion. Notch expression in HSCs is dependent on Wnt signaling and is necessary for maintenance of an undifferentiated state [69]. By using the transgenic Notch reporter (TNR.GFP^+^) mice, in which notch (expressed on ECs) signaling pathway stimulates GFP expression, ECs was confirmed to support long-term expansion of TNR.Gfp^+^cKit^+^Sca-1^+^Lineage^−^ (TNR.Gfp^+^KLS), but not Notch1^−/−^Notch2^−/−^CD34^−^Flt-3^−^KLS LT-HSCs. Also, upregulation of angiogenic expression of notch ligands shifts the balance between expansion and lineage-specific differentiation of the long-term HSCs toward expansion of LT-HSCs, decelerating differentiation [68]. Thus, within vascular niche, releasing angiocrine expressing notch ligands by ECs function to establish an instructive niche for the restoration of LT-HSC pool [45].

## 5. Vascular ECs-Secreting Factors Mediate Self-Renewal and Regeneration of HSCs

It has been mentioned above that the vascular niche has the ability to help HSCs self-renew and repopulate in BM, in which processes of ECs play crucial roles [33, 44]. Thus, BM EC-signaling is of vital importance in hematopoietic reconstitution* in vivo*. Himburg et al. and Kobayashi et al. suggested that soluble factors produced by BM ECs may be responsible for HSC self-renewal and regeneration* in vivo*, but the detailed mechanisms remain unknown [63, 70]. The mechanism of several factors will be discussed below.

### 5.1. Stem Cell Factor (SCF)

Stem cell factor (SCF) has been suggested to be expressed by endothelial cells, bone marrow fibroblasts, osteoblasts, CXCL12-expressing perivascular stromal cells, and nestin-expressing mesenchymal stem cells [71, 72]. Recently, it was reported that mice with the deletion of SCF expressed on endothelial cells (Tie2-cre; Scf^fl/−^) exhibit a decrease in LT-HSC frequencies with diminished repopulation capacities during congenic BM transplantation. SCF deletion in hematopoietic cells, osteoblasts, or Nestin^+^ cells did not alter HSC functions which identified SCF as a specific* in vivo* BM EC derived molecule that regulates HSCs [1].

### 5.2. Endothelial Selectins (E-Selectin)

Endothelial selectins (E-selectin) are cell adhesion molecules that are expressed at the vascular HSC niche to which hematopoietic stem and progenitor cells adhere [37]. Recent research revealed the adhesion molecule E-selectin expressed exclusively by bone marrow endothelial cells in the vascular niche. HSC quiescence was enhanced and self-renewal potential was increased in E-selectin knockout (Sele^−/−^) mice. Through administration of an E-selectin antagonist, the results demonstrated that E-selectin promotes HSC proliferation and is a crucial component of the vascular niche [37].

### 5.3. Glycoprotein 130 (gp130)

Glycoprotein 130 (gp130), a signaling subunit shared by interleukin-6 (IL-6) family of cytokines receptors, is also showed to make significant contribution to hematopoiesis. Deleting gp130 in hematopoietic and ECs of mice by the Cre/*loxP*-mediated gene recombination developed BM dysfunction resulting in severe anemia by adulthood. Normal hematopoiesis was reconstituted when transplanting gp130-deficient BM into irradiated wild-type mice, whereas hematopoietic defects still existed when transplanting wild-type BM into irradiated gp130-deficient mice. This result revealed that the expression of gp130 on the ECs rather than hematopoietic cells themselves has great influence on hematopoiesis, providing evidence that ECs depend on gp130 signaling to support hematopoiesis by responding to signals from IL-6 family cytokines [73].

### 5.4. Pleiotrophin (PTN)

A heparin-binding growth factor, pleiotrophin (PTN), secreted by BM ECs was recently shown to induce HSC regeneration in mice following high dose total body irradiation (TBI). These effects may be explained by binding and inhibition of the protein receptor tyrosine phosphatase-zeta (PTPRz) on BM HSCs [70].

## 6. Conclusion

Vascular niche plays a key role in supporting hematopoiesis including motility, transendothelial migration, and hematopoietic differentiation. What is more, compared to long-term dormant HSCs in osteoblastic niche, vascular niche keeps HSCs self-renewal, which leads to maintenance of HSCs. In conclusion, vascular niche has the potential to regulate hematopoiesis and maintain the self-renewal of HSCs. Thus reconstituting this niche in hematopoietic-disorder organism might replace the therapeutic method of HSCs transplantation. Endothelial therapeutic methods will spread light on hematopoietic diseases such as aplastic anemia and leukemia, speeding up the hematopoietic recovery after chemotherapy or shortening the time for hematopoietic reconstruction after BM transplantation. Although mechanistic pathways such as Notch and CXCR-4-SDF-1 signaling have been shown to contribute to BM endosteal and reticular cell regulation of HSCs fate* in vivo*, the mechanisms through which vascular niche regulate HSCs maintenance and regeneration remain less well defined. And elucidation of the mechanistic cross-talk and coregulation of hematopoiesis by osteoblasts, ECs, and other BM microenvironment cells will be a central objective of the coming decade. Further research will push the existed results forward and make great breakthrough on vascular niche.

## Figures and Tables

**Table 1:** 

Molecules	Function	References
FGF-4	Supports the adhesion of megakaryocytes to BMECs	[34, 65]
SDF-1	Induces the transendothelial migration and plays a key role in HSCs differentiation	[58, 59]
VCAM-1	Induces the expression of adhesion molecules	[34, 65]
G-CSF	Causes HSC mobilization	[66]
Tie2	Mediates HSCs to adhesion to osteoblasts, regulating the quiescence in osteoblastic niche	[14]
SCF	A key niche component that maintains HSCs	[71]
E-selectin	Enhances HSC quiescence and self-renewal potential and promotes HSC proliferation	[37]
gp130	Makes significant contribution to hematopoiesis	[73]
PTN	Induces HSC regeneration	[70]
